# An open trial assessment of "The Number Race", an adaptive computer game for remediation of dyscalculia

**DOI:** 10.1186/1744-9081-2-20

**Published:** 2006-05-30

**Authors:** Anna J Wilson, Susannah K Revkin, David Cohen, Laurent Cohen, Stanislas Dehaene

**Affiliations:** 1 INSERM-CEA Unit 562 « Cognitive Neuroimaging » Service Hospitalier Frédéric Joliot, CEA-DRM-DSV, 91401 Orsay, France; 2 Collège de France, 11 place Marcelin Berthelot, 75231 Paris Cedex05, France; 3 Service de Neurologie, Hôpital de la Pitié-Salpêtrière, AP-HP, 47 bd de l'Hôpital, 75013, Paris, France; 4 Department of Child and Adolescent Psychiatry, Université Pierre et Marie Curie, Laboratoire CNRS "Du comportement et de la cognition", Hôpital Pitié-Salpêtrière, AP-HP, 47 bd de l'Hôpital, 75013, Paris, France

## Abstract

**Background:**

In a companion article [1], we described the development and evaluation of software designed to remediate dyscalculia. This software is based on the hypothesis that dyscalculia is due to a "core deficit" in number sense or in its access via symbolic information. Here we review the evidence for this hypothesis, and present results from an initial open-trial test of the software in a sample of nine 7–9 year old children with mathematical difficulties.

**Methods:**

Children completed adaptive training on numerical comparison for half an hour a day, four days a week over a period of five-weeks. They were tested before and after intervention on their performance in core numerical tasks: counting, transcoding, base-10 comprehension, enumeration, addition, subtraction, and symbolic and non-symbolic numerical comparison.

**Results:**

Children showed specific increases in performance on core number sense tasks. Speed of subitizing and numerical comparison increased by several hundred msec. Subtraction accuracy increased by an average of 23%. Performance on addition and base-10 comprehension tasks did not improve over the period of the study.

**Conclusion:**

Initial open-trial testing showed promising results, and suggested that the software was successful in increasing number sense over the short period of the study. However these results need to be followed up with larger, controlled studies. The issues of transfer to higher-level tasks, and of the best developmental time window for intervention also need to be addressed.

## Background

In the preceding accompanying article [1], we described the development and validation of software designed to remediate dyscalculia. In this article, we first put the use of the software in its context with a discussion of dyscalculia including its symptoms, causes, and possible subtypes. We then present results from initial testing of this software in an actual remediation setting, using a group of children with mathematical learning difficulties.

### Developmental dyscalculia

Developmental dyscalculia ("dyscalculia") is a disorder in mathematical abilities presumed to be due to a specific impairment in brain function [2-4]. This neuropsychological definition is highly similar to legal definitions of mathematical disabilities (e.g. Public Law 94–142 in the United States). However although the theoretical definition of these constructs is generally agreed upon, their operalization is another issue, and varying selection criteria have tended to result in considerable differences between the populations in different studies [5]. This is essentially because much remains to be discovered about the symptoms of dyscalculia; in fact research on dyscalculia is in its infancy compared to research on its reading analog, dyslexia. Here we briefly review what is currently known. For a more in-depth discussion of this complex topic, we refer the reader to a recent chapter [6], as well as to several excellent reviews [5,7-10].

Dyscalculic children show a variety of fundamental mathematical deficits. These include an early delay in understanding some aspects of counting [11-13], and a later delay in using counting procedures in simple addition [12,14-16]. They also include a persistent deficit in memorizing and recalling arithmetic facts (eg. 3 + 7 = 10 or 4 × 5 = 20) [16-23]. In addition, recent studies (discussed further below) have suggested that dyscalculic children show numerical deficits at an even lower level, that of the representation of quantity and/or the ability to link quantity to symbolic representations of number.

The causes of dyscalculia remain unknown. Several researchers have argued for a genetic component [24]. Indeed, dyscalculia is frequently observed in several genetic disorders such as Turner's syndrome [25], Fragile X syndrome [26], and Velocardiofacial syndrome [27]. However, factors such as premature birth and prenatal alcohol exposure are also associated with higher rates of dyscalculia [28,29]. Co morbid disorders are common, particularly attentional deficit hyperactivity disorder (ADHD), and dyslexia [17,19,21].

#### Proposed subtypes of dyscalculia

It has been proposed by many authors that there are subtypes of dyscalculia, resulting from different causes and showing different symptom profiles. However the evidence on this question remains inconsistent. A full discussion of this is beyond the scope of the current paper; however it should be noted that much work has been carried out addressing this issue in the special education field [for reviews consult [7,8,24,30]].

Two recent subtype proposals are those of Geary [8,24], and of Jordan and colleagues [16,31-33]. Based on a review of the cognitive, neuropsychological and genetic literature, Geary [24] proposed that there are three subtypes of dyscalculia. The first is a procedural subtype, due to executive dysfunction and characterized by a developmental delay in the acquisition of counting and counting procedures used to solve simple arithmetic problems. The second is a semantic memory subtype, due to verbal memory dysfunction and characterized by errors in the retrieval of arithmetic facts. This type is linked to phonetic dyslexia. These first two subtypes fit fairly well with observed symptoms of dyscalculia. The third proposed subtype is due to visuospatial dysfunction; however while this subtype is found in adult acquired dyscalculia, evidence for it in developmental dyscalculia is scarce, because few studies have examined dyscalculic children's spatial abilities. (One exception is a recent study by Mazzocco and colleagues [34]).

Jordan and colleagues have also argued for a subtype linked to dyslexia, and have conducted several studies revealing that children who have co-morbid dyslexia (MDRD, for math and reading disabilities) show a different pattern of deficits in mathematics than those who have pure dyscalculia (MD only) [16,31-33]. However, not all studies have found different profiles for these groups on basic numerical cognition tasks [e.g. [35,36]]. In addition the differences found are only a single dissociation (MDRD children perform worse than MD only children on word problems and untimed fact retrieval), leaving open the possibility that MDRD children are simply have more difficulties in general, thus showing a quantitative rather than a qualitative difference. Jordan and others have also conducted studies suggesting that children showing "fact retrieval deficits" might form a particular subtype [37,38], consistent with Geary's semantic memory subtype. Thus these two subtype proposals are not mutually exclusive.

#### Number sense

Up until very recently, most research on the symptoms of dyscalculia focused on higher level tasks such as addition, subtraction and problem solving. The problem with this is that multiple cognitive processes are likely to contribute to each of these tasks, and thus they may not be ideal for clearly illuminating key symptoms of dyscalculia. Contrary to this approach, research in normal adult numerical cognition has focused on basic component processes and used extremely simple tasks. This research has led to the identification of a core aspect of numerical cognition: "number sense", or the ability to represent and manipulate numerical quantities non-verbally [39,40]. (Note that the phrase "number sense" is also used in the special education community with varied and broader meanings, for a discussion see [41]. Here we used the phrase as used in the cognitive neuroscience literature [39]).

Number sense is known to be present within the first year of life, and to undergo normal development during early childhood, in particular a progressive refining of the accuracy with which numerical quantities can be estimated [42,43]. Number sense does not depend on language or education, inasmuch as it is present in Amazonian children and adults without formal education or a developed number lexicon [44]. This ability has now been linked to a particular area of the brain, the horizontal intra-parietal sulcus (HIPS) in both adults and children [45-47].

#### Dyscalculia as a core deficit in number sense

Could dyscalculia, or at least one of its subtypes, be the result of an impairment in number sense? Recently, several studies have tested dyscalculic children's performance on the same tasks used in adult numerical cognition research. For instance Landerl et al. [35] found that dyscalculic children were slower at numerical comparison (but not non-numerical comparison) compared to controls, and that they showed deficits in subitizing (rapid apprehension of small quantities). These results confirmed a sole earlier finding that dyscalculic children were slower to process numbers (compared to letters) relative to non-dyscalculic children, and that they appeared to be slower in subitizing [48]. Rousselle and Noël [36] also found that dyscalculic children were slow to perform number comparison, and that some subjects lacked the typically observed "distance effect" (higher reaction times for smaller numerical distances). Some evidence also suggests that dyscalculic children may exhibit less automatic activation of quantity from Arabic digits [49] (although other authors have failed to replicate this result [36]).

Other studies have started to look at the predictive value of number sense measures, which have recently been proposed for use in screening for dyscalculia, based on cross-sectional correlations with mathematics performance in kindergarten and first grade [50-53]. For instance Mazzocco and Thompson [53] showed that kindergarteners who perform badly on number comparison, number constancy, and reading numerals are likely to show persistent dyscalculia in grades 2 and 3.

As of yet, the neural bases of dyscalculia have only been investigated in special populations, but results from these studies reveal abnormalities in the area associated with number sense. For instance, one study showed that dyscalculic adolescents who were born pre-term had less grey matter in the left HIPS than non-dyscalculic adolescents who were born pre-term [28]. Molko and colleagues [25,54] found that young adult women with Turner's syndrome, which is associated with dyscalculia, showed structural and functional abnormalities in the same area, particularly in the right hemisphere.

Based on this data, we and others have proposed that dyscalculia (or at least one of its subtypes) may be caused by a core deficit, this deficit being either in number sense itself, or in its access from symbolic number information [6,41,52,55-57]. (Note: Although we refer to both of these hypotheses as falling under a "core deficit", other authors have termed the latter hypothesis the "access deficit" hypothesis [36].) According to the triple-code model of numerical cognition [58,59], numbers are represented in three primary codes: visual (Arabic digits), verbal (number words), and analog (magnitude representation). The non-symbolic (analog) representation appears to develop early in infancy [42], but children establish the symbolic representations as a result of culture and education [36,44]. An essential aspect of the triple-code model is the presence of bidirectional transcoding links between all three representations. Dyscalculia might therefore be caused by either a) a malfunction in the representation of numerical magnitude itself, or by b) malfunction in the connections between quantity and symbolic representations of number.

These two hypotheses could be separated based on performance on non-symbolic tasks. Direct impairment of the quantity system would result in failure on both non-symbolic and symbolic numerical tasks, whereas a disconnection should leave purely non-symbolic tasks intact. A recent study [36] brought support to the disconnection hypothesis, with dyscalculic children showing a deficit only on symbolic number comparison and not on non-symbolic comparison. However, this issue needs to be investigated further.

#### How to evaluate dyscalculia?

In spite of this remaining uncertainty, the core deficit hypothesis readily leads to a choice of tests that might be appropriate to reveal symptoms of developmental dyscalculia. These symptoms should be similar to those seen in cases of acquired acalculia where number sense is impaired. They might therefore include a reduced understanding of the meaning of numbers, and a low performance on tasks which depend highly on number sense, including non symbolic tasks (e.g. comparison, estimation or approximate addition of dot arrays), as well as symbolic numerical comparison and approximation.

With respect to simple arithmetic, we would expect that subtraction would provide a more sensitive measure of a core number sense deficit than either addition or multiplication. This is because addition and particularly multiplication problems are thought to be more frequently solved using a memorized table of arithmetic facts than are subtraction problems. In adult acalculic patients, subtraction can double-dissociate from addition and multiplication: subtraction deficits are frequently associated with impairment in number sense tasks such as approximation, while addition and especially multiplication deficits are frequently associated with aphasia or alexia [40,46,60,61]. If these findings can be generalized in a developmental context, they would suggest that dyscalculic children of the "core number sense" subtype should show particular difficulties with elementary subtraction problems.

Based on the adult neuropsychological data, we would expect that more verbal tasks such as counting and fact retrieval should be less affected. However, this latter prediction is not clear-cut because it is unknown to what extent number sense contributes to the acquisition of counting principles (especially cardinality) and helps bootstrap an early understanding of the meaning of addition and subtraction. Even though fact retrieval in adults relies largely on a rote memory process, it is possible that number sense could aid in retrieval success by increasing the semantic content of the information being retrieved [62].

The previously discussed low-level symptoms observed in dyscalculia (impairments in speed of processing of numerical information, in numerical comparison and subitizing, and possibly in automatic access to magnitude information) are all consistent with the core deficit theory. The higher level symptoms (impairments in acquisition of counting and addition procedures and in fact retrieval) may be a derivative of an initial dysfunction of the core number sense system. However there are many other possible causes of these high-level symptoms. One way to indirectly test the core deficit theory is to test the response of children to a primarily quantity-based intervention, an undertaking which we present in the current study.

### "The Number Race" software

In the accompanying article [1], we described in detail the development and validation of "The Number Race", an adaptive game designed for the remediation of dyscalculia. The software was designed with both of the possible causes of a core deficit in mind. In order to enhance number sense, it provides intensive training on numerical comparison and emphasizes the links between numbers and space. In order to cement the links between symbolic and non-symbolic representations of number, it uses scaffolding and repeated association techniques whereby Arabic, verbal and quantity codes are presented together, and the role of symbolic information as a basis for decision making is progressively increased. In addition, higher levels of the software are designed to provide training on small addition and subtraction facts, although this training is restricted to a small range of numbers and provides conceptually oriented, concrete representations of operations rather than drilling of arithmetic facts.

#### The current study

The current study was carried out as the first step in an ongoing series of tests of the efficacy of the "Number Race" software. In this first study, we used an open-trial design, analogous to the first stage of testing of a new medical therapy: We identified a group of children who had learning difficulties in mathematics, and tested their performance on a battery of numerical tasks before and after remediation. This design had two primary goals: 1) to determine whether performance improves significantly between the pre- and post-training periods, a minimal requirement before proceeding with larger and more expensive studies; and 2) to identify which measures of arithmetic performance are most sensitive to training.

An analysis of children's improvement profiles across the tasks tested at pre and post remediation also allowed for an assessment of the coherence of this pattern with the core deficit hypothesis. The tasks tested were based on previous work in adults with acquired acalculia as well as on previous work in developmental dyscalculia. They covered the main cognitive processes involved in numerical processing as well as the basic academic skills relevant to schoolwork in early elementary school. They included verbal counting, transcoding, base-10 comprehension, enumeration (permitting measurement of subitizing and counting), addition, subtraction, and symbolic and non symbolic number comparison. We hypothesized that children would show the largest improvement on tasks which draw more heavily on number sense, such as number comparison, subtraction, and to a lesser extent, addition. Conversely, we did not expect to see much improvement in counting or transcoding, because these tasks do not depend much, if at all, on number sense. If a core deficit is caused by difficulties in linking symbolic and non-symbolic information, we should see greater improvement in symbolic rather than non-symbolic number comparison. Many more specific patterns could be expected within particular tasks based on research in normal subjects and adult acalculic patients; we discuss these below in the context of each task.

## Methods

### Sample

Twenty two French children aged 7–10 years were recruited from three participant schools in Paris by teacher recommendation; which was based on the observation of persistent and/or severe difficulties in mathematics. We carried out exclusion screening for these children, of whom 13 were selected for the study. All children and their families gave informed consent prior to screening.

Children were tested by a native French speaker and trained neuropsychologist using a WISC-III [63] short form [64] consisting of vocabulary, picture completion, and arithmetic. Children were excluded if they had an estimated IQ of less than 80 using the non-arithmetic WISC-III subtests (3 children), lack of a below average score on the WISC arithmetic (2 children), or behavioral difficulties (1 child). Three children were excluded due to diverse other reasons (teacher withdrawal, failure to meet age requirements, and visual problems). Four of the children who participated in the study were eventually excluded from the final sample due to extended absences (two children), disruptive behavior (one child), and the discovery that French was not the child's first language (one child).

The final sample for the study was thus nine children between the ages of 7 and 9 (average age = 8.1 years). Of the final sample, three of the children were repeating a school year (this is common practice in France for children who are not making adequate progress). The arithmetic subtest scores of the WISC for the final sample ranged from 1^st ^to 37^th ^percentile, with the average score at the 12^th ^percentile, confirming children's low mathematics performance. It should be noted that in the absence of recruitment from a large population and the use of a strict cut-off procedure, the current sample is best described as children with mathematical learning difficulties rather than dyscalculia *stricto sensu*.

### Procedure

The study took place at school during school hours over a period of 10 weeks. Screening and pre-testing occurred in the first two weeks, children were on vacation during the next two weeks, and then completed their remediation in the fifth to ninth weeks. This consisted of one half-hour session using the software for each child four days a week, supervised by the authors (AJW & SKR), thus for a maximum of ten hours (due to absences, the average was eight hours). During the tenth week, the children were post-tested. (One child fell ill during this period, and had to be tested three weeks later.)

### Testing battery

Children were tested in three half-hour sessions, primarily using a computerized testing battery. Tasks included were enumeration, symbolic and non-symbolic numerical comparison, addition, and subtraction. These tasks were designed to measure basic components of numerical cognition, and were based on work in adult acalculic patients [61], as well as in recent work in developmental dyscalculia [35]. We supplemented the computerized battery with three non-computerized tasks (counting, transcoding and understanding of the base-10 system), which were sub-tests drawn from the TEDI-MATH battery [65]. This battery was developed for the assessment and profiling of dyscalculia.

#### Non-computerized tasks

The three non-computerized tests took a half-hour to complete and were administered by a native French speaker and trained neuropsychologist. Because the TEDI-MATH battery is not available in English we describe them briefly below.

##### Counting

The test included 6 items, each worth 2 points. Children were asked to count as high as they were able (the experimenter stopped them at 31), to count up to particular numbers (9 and 6), to count starting at particular number (3 and 7), to count from one number to another number (5 to 9 and 4 to 8), to count backwards from a number (7 and 15), and to count by 2 s and by 10 s.

##### Transcoding

This test was designed to measure children's ability to read and write Arabic digits. In the first part of the test, children were dictated 20 numbers, ranging from 1 to 3 digits, which they wrote on a sheet of paper. In the second part of the test, children were asked to read 20 written Arabic numbers, also ranging from 1 to 3 digits.

##### Base-10 comprehension

In the first part of this test (11 points), children were shown small plastic rods arranged in bundles of ten, as well as a stack of individual rods. The tester showed three combinations of rods and bundles and the child had to say how many rods were in each combination (20, 24, and 13). Then children were given a verbal quantity and asked how many bundles and rods would be needed to make this quantity (14, 20, 8, and 36). Finally they were told that the tester had a certain quantity of rods, and wanted to give another quantity to a friend; would she need to break open a bundle to do this? (15-7, 29-6, 16-5, and 32-4).

In the second part of the test (6 points), children were given two types of round tokens, which they were told represented money (1 euro and 10 euros). They were asked to show how many tokens would be required to buy a toy which cost a particular amount (17, 13, 19, 23, 15 and 31 euros).

Finally, in the third part of the test (10 points), children were shown a sheet of paper with written numerals, and asked to circle the ones column (28, 13, 10, 520, and 709) or the tens column (20, 15, 37, 650, 405).

#### Computerized tasks

The computerized tasks were administered in two half-hour sessions. In the first session, children completed dot enumeration, addition and subtraction. In the second session they completed the two comparison tasks (symbolic and non-symbolic). Children were given instructions at the beginning of each task, and then completed several training trials (using different stimuli from experimental trials where possible). The experimenter sat next to the child throughout the task to ensure that they were paying attention, and gave the child breaks of several min as needed.

All tasks were presented on a Celeron laptop running E-Prime software [66], with a 14 inch screen set to 600 × 800 pixel resolution. We measured accuracy and reaction time for all tasks. For those requiring a voice response, reaction time was measured using a microphone connected to a serial response box, and the child's responses were recorded by the experimenter, who also coded for microphone errors. If children had difficulties with the microphone, a back-up system was used, in which the experimenter pressed a key as soon as they responded. In tasks requiring a manual response, children pressed one of the two touch-pad mouse buttons to indicate the side of the screen (left or right) of the correct stimulus. Children were given no feedback on their accuracy. In all tasks children had to respond within 10 sec (except in addition and subtraction, for which they had 15 sec).

##### Dot enumeration

In this task, based on Mandler and Shebo [67], we examined children's subitizing and counting performance by measuring verbal reaction times for enumeration of sets of one to eight dots. Children were told to count whenever they needed to. They completed 64 trials in two blocks, which took around 10 min. Stimuli consisted of 64 square 350 by 350 pixel white images, each containing a set of 1 to 8 randomly arranged black 36 pixel diameter dots (8 images for each numerosity 1 through 8). They were generated using a Matlab program which generated dot displays controlled for overall occupied area (thus distance between the dots was larger for smaller numerosities). The time course of each trial was as follows: The trial began with an auditory alerting signal (a beep) concurrent with the appearance of a visual alerting signal (a green fixation symbol composed of the two signs <> presented in the centre of the screen). The dot display appeared in the centre of the screen 1500 msec after this signal, and remained on screen for 10 sec, or until the child responded. The screens had a black background throughout the experiment. As soon as the child responded they were presented with a "reward" image for one second, which was an attractive square tile filled with abstract color patterns. The same image appeared on all trials, regardless of the accuracy of the response. After the offset of this image, there was a two second inter-trial interval.

A normal pattern of results is a reaction time curve which is almost constant over the numerosities 1–3 (reflecting subitizing), and then increases steadily for numerosities 4–8 (reflecting counting). Previous work suggests that dyscalculic children show slowing in both the subitizing and counting range, although more markedly in the subitizing range [35,48]. In addition, in adult acquired acalculia patients, subitizing deficits have been associated with number sense deficits [61]. We thus might expect that children's performance in both of these ranges would improve after remediation, but more so in the subitizing range, which provides the purest measure of quantity processing.

##### Addition

In this task we measured verbal responses to single digit addition problems. Children were told they could use their fingers if they needed to. They completed 32 trials in two blocks, which took around 10 min. Each problem was coded by type, which had four categories: tie, rule, normal-small, and normal-large. Tie items consisted of problems with identical operands (e.g. 5 + 5). Rule items consisted of sums which can be solved by the application of a simple rule, in this case x + 0 = x. Normal items were items which did not fall in either of these categories. These were divided into two groups according to problem size, which had two categories: "small problems" (sum 10 and under) and "large problems" (sum 11 or over). Stimuli consisted of 32 single digit addition problems, with the larger digit always listed first. The stimulus selection process was thus as follows: all possible single digit addition pairs were listed, and coded for type and magnitude. A full cross of the factors of type and magnitude was possible, except that for the type "rule", there were no large problems. The cell size for each type was reduced so that there were 16 normal problems (8 small, 8 large), 8 rule problems, and 8 tie problems (4 small, 4 large). The time course of each trial was the same as in the dot enumeration task, except that children had 15 sec to respond. Problems were presented in 18 point courier font and colored white.

A normal pattern of results is for rule (ie. x + 0 = x) and tie (ie. x + x = y) items to show faster response times, and for small items to show faster response times than large items ("magnitude effect", see [68] for a historical review). As previously mentioned, dyscalculic children tend to show a developmental delay in the use of addition procedures involving counting and also in recall of addition facts. Addition procedures such as finger counting, or counting up from the larger addend, were not included in the software, nor was practice on facts with a sum larger than ten. The software did of course emphasize understanding and manipulation of quantities, and thus to the extent that addition involves this, we might expect children to show some improvement. However, the role of number sense in addition is not clear-cut. In adults at least, exact addition appears to involve primarily rote memory processes. Thus it was not clear whether children would show improvement in addition or not.

##### Subtraction

In this task we measured verbal responses to single digit subtraction problems. Children were told they could use their fingers if they needed to. They completed 36 trials in two blocks, which took around 10 min. There were three types of problem: "rule", "small subtrahend" and "large subtrahend". Rule items consisted of items which can be solved by the application of a simple rule, such as x – 0 = x, or x – x = 0. All other items were items which did not fall in this category. Small subtrahend items had a subtrahend of 2–4 (inclusive) and large subtrahend items had a subtrahend between 5–8 (inclusive). Stimuli consisted of 36 single digit subtraction problems, with the larger digit always listed first. The stimulus selection process was as follows: all possible single digit subtraction pairs were listed, and coded for type (rule vs. non-rule and small vs. large subtrahend). The cell size for each type was reduced so that there were 18 normal problems (10 small subtrahend, 8 large subtrahend), and 18 rule problems. The time course of each trial was the same as in the addition task. Problems were presented in 18 point courier font and colored white.

A normal pattern of results is for rule items (x – 0 = x, or x – x = 0) to show faster response times (because quantity manipulation is not required to resolve them), and for items with a large subtrahend to show slower reaction times than those with a small subtrahend (reflecting counting backwards, or counting up from the subtrahend). As previously mentioned, dyscalculic children also show a developmental delay in the use of subtraction procedures involving counting. Unlike addition, however, in adults subtraction (of non-rule items) is thought to depend primarily on the ability to understand, represent and manipulate quantity [46]. Therefore we would expect children to show considerable progress in subtraction (at least on non-rule items).

##### Numerical comparison: symbolic

In this task, based on Moyer and Landauer [69], children were presented with two digits of different numerical sizes, and had to indicate which was the largest. Children completed 36 trials in two blocks, which took around 5 min. Stimuli consisted of all of the possible pairs of one digit numbers (excluding zero) irrespective of order, giving a total of 36 pairs. The side of the largest number was varied randomly from trial to trial. The time course of each trial was the same as in the dot enumeration task. The pairs of digits were presented in white on a black screen, offset 40 pixels from fixation.

A normal pattern of results is the classical "distance effect", in which reaction times are longer and accuracy lower to compare numbers which are closer than numbers which are further away [69]. As previously mentioned, dyscalculic children are slower at symbolic number comparison than non-dyscalculic controls [35]. Given that number comparison is the primary task in the software, at the least we expected children to show a general increase in performance. Furthermore, we expected children to show a change in the shape of the distance effect, reflecting an increase in precision of quantity representation.

##### Numerical comparison: non-symbolic

In this task, children were presented with two arrays of dots of two different numerosities, and had to indicate which was the largest. The numerosities used were exactly the same as those used in the symbolic task. Children completed 36 trials in two blocks, which took around 5 min. Stimuli were 250 pixel diameter white circles, containing arrays of black dots. All possible pairs of one digit numerosities (excluding zero) were included, giving a total of 36 pairs. Half of the stimuli pairs were equalized for total occupied area (of the array) and dot size, but varied in total luminance and density, and the other half were equalized for total luminance and density, but varied in total occupied area and dot size. Dot arrays presented in a given run of the experiment were randomly drawn (without replacement) from a pool of arrays double the size needed. The side of the largest number was varied randomly from trial to trial. The time course of each trial was the same as in the symbolic comparison task, except that in order to avoid counting, the pair of dot arrays were flashed on screen for only 840 msec, in conjunction with a central yellow fixation star (*), which then remained on-screen for 9160 msec, or until the child responded. Stimuli were offset 130 pixels from fixation.

The interest of including this task was to compare children's progress on it to that on the symbolic task. If a core deficit is caused primarily by difficulties in linking symbolic and non-symbolic information, we should see an improvement in symbolic number comparison, but considerably less improvement in non-symbolic number comparison.

## Results

For all computerized tasks, mean accuracy and median reaction time (RT; for correct responses only) were calculated for each subject within each condition. These values were then entered in repeated measures ANOVAs, to compare pre and post differences. We tested for all main effects and interactions.

### Non-computerized tasks

Children showed marginally significant improvements in the counting subtest of the TEDI-MATH, with an average pre and post scores of 9/12 and 11/12 respectively (t(8) = 2.24, p = 0.056). They also showed a small significant improvement in transcoding, with average pre and post scores of 36/40 and 39/40 respectively (t(8) = 2.42, p = 0.04). In both of these tasks about half the children were below their age and/or class level at pre-testing, and all had reached at least their class level at post testing. However understanding of base-10 system showed no improvement; even though four children were considerably below age-level at pre-testing.

### Computerized tasks

#### Dot enumeration

**Figure 1:**
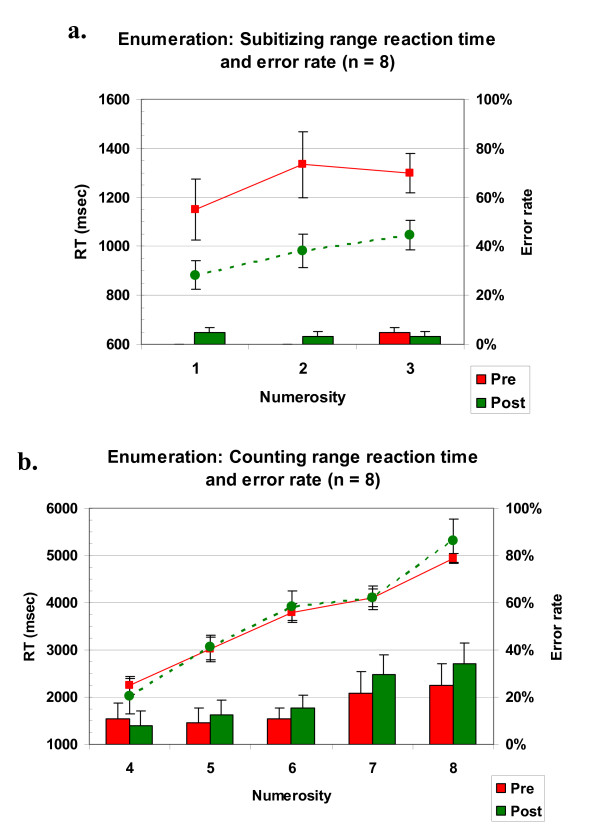
**Enumeration performance in the subitizing and counting ranges**. a) Average enumeration reaction times and accuracy in the subitizing range, showing a significant improvement (p = 0.003) at post-test. b) Average enumeration reaction times and accuracy in the counting range. No difference is shown between pre and post-test.

Dot enumeration results are shown in Figures 1a (subitizing range) and 1b (counting range). Results showed a large improvement in speed for the subitizing range but not the counting range. Following previous authors [35], we analyzed the data in two separate ANOVAs, a 2 × 3 ANOVA for the subitizing range, and a 2 × 5 ANOVA for the counting range. Data from one subject was excluded from both of the analyses, because she showed a highly abnormal pattern at post-testing, and because 5 out of her 6 medians in the subitizing range were outliers in the group distribution (a distance of over 2 times the inter-quartile range from the median). In the subitizing range, children's RT decreased an average of nearly 300 msec in the second session relative to the first, and the RT analysis showed significant main effects of session (F(1,7) = 19.1, p = 0.003), and of numerosity (F(2,14) = 6.18, p = 0.01). There was no significant session x numerosity interaction. Children were slightly less accurate in the second session (96% in the second session vs. 98% in the first), however this difference was non- significant (F(1,7) = 2.33, p = 0.17), thus it is unlikely that it indicates a speed/accuracy trade-off. In the counting range, children's overall performance showed almost no change. Both the RT and accuracy analyses revealed only highly significant main effects of numerosity (F(4,28) = 66.2, p < 0.001, F(4,28) = 4.65, p = 0.005, respectively), but no change in the mean or slope of performance as a function of numerosity.

#### Addition

Addition results are shown in Table 1. The data were analyzed using 2 × 4 ANOVAs (session × problem type). The main effect of session was not significant for either accuracy or RT, which stayed at a similar level from pre to post testing. The main effect of problem type was significant in both the accuracy and RT analyses (F(3,24) = 14.9, p < 0.001; F(3,24) = 92.7, p < 0.001, respectively), however this simply reflected normal differences between rule and tie vs. "normal" problems, as well as a magnitude effect (worse performance with larger operands). This effect did not show an interaction with session, and no improvement was seen in any of the four categories of problems.

**Table 1:** Mean accuracy and reaction time for addition task, and reaction time for subtraction task.

	Rule	Tie	Small	Large
Addition: Accuracy (%)				
Pre	100 (0)	76 (5)	92 (2)	61 (10)
Post	97 (3)	88 (5)	89 (2)	64 (9)
Reaction Time (msec)				
Pre	1995 (245)	2974 (293)	3172 (365)	6892 (347)
Post	1694 (229)	2908 (618)	3244 (368)	6427 (454)

Subtraction: Reaction Time (msec)				
Pre	2564 (172)		6032 (512)	8010 (834)
Post	2428 (179)		6724 (725)	8575 (1049)

Note. Parentheses contain standard error.

#### Subtraction

**Figure 2:**
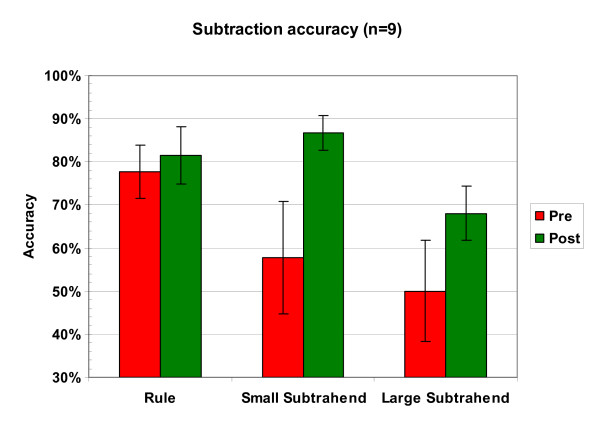
**Performance in subtraction before and after training**. Subtraction average accuracy (significant main effect of session, p = 0.04). "Rule" items were items such as x-x = 0 or x-0 = x. Small subtrahend items had a subtrahend from 2–4 inclusive, and large subtrahend items had a subtrahend from 5–8 inclusive.

Subtraction accuracy is shown in Figure 2, and reaction time in Table 1. Children's performance, which was initially low, showed a large pre-post change. The data were analyzed using 2 × 3 ANOVAs (session × problem type). In the accuracy analysis, the main effect of session was significant (F(1,8) = 6.51, p = 0.03), and the main effect of problem type was highly significant (F(2,16) = 6.85, p = 0.007). The overall interaction between session and problem type was not significant, however the largest improvements were seen in the non-rule-based problems: accuracy increased to from 58% to 87% for small subtrahend problems (p = 0.07 using a post-hoc t-test) and from 50% to 67% for large subtrahend problems (p = 0.08), while performance on rule problems, in contrast, showed no significant change across session. In the RT analysis (Table 1), only a highly significant effect of problem type was observed (F(2,12) = 28.9, p < 0.001). (Note: 4 out of 54 observations in the analysis had missing data, due to the lack of any correct responses available to contribute to the median reaction time.)

#### Number comparison: symbolic

**Figure 3:**
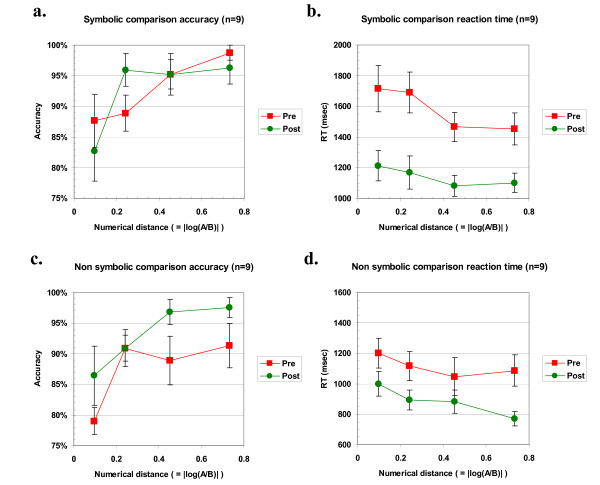
**Performance in symbolic and non-symbolic comparison before and after training**. a) Symbolic comparison (Arabic digit) accuracy, plotted as a function of the distance between the numbers (measured by their log ratio). A slight change in shape in the post curve suggests an increase in precision of the quantity representation. b) Symbolic comparison (Arabic digit) reaction time. A significant decrease is seen between pre and post curves in overall RT (p = 0.002). c) Non-symbolic comparison (dot clouds) accuracy. A significant increase in accuracy at post test (p = 0.01) suggests a more precise representation of numerosity. d) Non-symbolic comparison (dot clouds) reaction time. A significant decrease is seen in overall RT (p = 0.006). Note: Error bars indicate one standard error.

The symbolic number comparison data are shown in Figures 3a and 3b, and were analyzed by 2 × 4 (session × distance) ANOVAs. For each trial, the numerical distance between the two stimuli (A and B) was evaluated as their log ratio |log(A/B)|. Trials were grouped into four categories of increasing distance, maintaining roughly equal cell sizes. Graphs show the (weighted) mean Weber fraction for each category. The accuracy analysis showed no significant main effect for session, although as was expected there was a highly significant main effect for distance (F(3,24) = 7.51, p = 0.001). The session × distance interaction fell short of significance (F(3,24) = 2.41, p = 0.09). As can be seen from Figure 3a, this reflects a slight change in the shape of the accuracy curve from pre to post testing, consistent with the post-testing curve becoming steeper. This suggests that the precision of children's numerical representation may have increased.

In addition, children were overall much faster in responding at post test (by 468 msec), reflected in a significant main effect of session (F(1,8) = 19.7, p = 0.002) in the RT analysis. The main effect of distance was also significant (F(3,24) = 7.08, p = 0.001). Children tended to show slightly less of a change in RT across distance in the post test, although this interaction did not reach significance (p = 0.17). This trend is similar to changes which occur as a result of normal development [71], which are an overall increase in speed and a reduction in the slope of the distance effect.

#### Number comparison: non-symbolic

The non-symbolic number comparison data were analyzed in exactly the same way as the symbolic comparison data and are shown in Figures 3c and 3d. Children's initial performance on this task was less accurate overall than in the symbolic task, but at post testing there was an overall increase in accuracy (+5%), reflected in a significant main effect of session (F(1,8) = 9.81, p = 0.01), again suggesting a higher precision after training. The main effect of distance was highly significant (F(3,24) = 6.70, p = 0.002). The session × distance interaction was not significant.

Children's initial speed of response for this task was much faster than for the symbolic comparison task. Nevertheless, they still showed a significant speed increase of 226 msec across the remediation period (main effect of session significant, F(1,8) = 13.8, p = 0.006). The main effect of distance was also significant (F(3,24) = 6.56, p = 0.002). There was no significant interaction, reflecting the fact that the distance effect was around the same size at pre and post testing.

In order to test whether the effects of remediation were different for the two types of comparison tasks (symbolic vs. non-symbolic), we ran post-hoc 2 × 2 × 4 (task × session × distance) ANOVAs for both reaction time and accuracy. For reaction time, a main effect of task was observed (F(1,8) = 19.24, p = 0.002), reflecting the fact that children were faster on average at the non-symbolic task. There was a significant task × session interaction (F(1,8) = 6.77, p = 0.03), reflecting the larger improvement in reaction time for the symbolic comparison task. For accuracy, however, there was a trend towards a task × session interaction (F(1,8) = 3.99, p = 0.08) but in the converse direction, reflecting a larger increase in accuracy for the non-symbolic task. This limits interpretation of the task × session interaction in reaction time, because it might be due to a speed-accuracy trade-off. In the accuracy analysis, the task × session × distance interaction was significant (F(3,24) = 3.17, p = 0.04), reflecting an increase in accuracy for the non-symbolic task at far distances, whereas the symbolic task showed an increase only at one close distance.

## Discussion

Children's results from pre and post testing showed improvement on several tasks, suggesting that the remediation was successful in producing an improvement in basic numerical cognition. Of course the use of an open-trial design limits the conclusions which can be made, due to the lack of a control group. It is important for future studies to allow a separation of which effects are due to the software, and which can be attributed to general attentional or motivational improvements, practice with computers, test-retest effects, or regression to the mean. Notwithstanding, we argue that many of the specific improvements seen are unlikely to be due to such general factors. For example, in order to propose that the increase in enumeration speed was the result of general attentional improvements or regression to the mean, one would have to explain why it only occurred within a specific range of small numerosities 1–3.

### Consistency of results with the core deficit hypothesis

To what extent are the results observed consistent with the core deficit theory of dyscalculia? As predicted, children showed improvements in performance on classical number sense tasks. This was most marked in numerical comparison (both symbolic and non-symbolic), in which performance was 200–450 msec faster at post test. There were also some indications that in the symbolic task the quantity information extracted by children might have become more precise: in accuracy the distance effect tended towards a steeper curve, and in reaction time the distance effect showed a trend towards being smaller at post test. A similar effect was not seen for the non-symbolic task. The latter results seem consistent with the idea that a core deficit might be caused by impairment in the links between non-symbolic and symbolic representations; however they should be interpreted with caution since they were only marginally significant.

Results from the enumeration task supported the core deficit hypothesis, with children's speed of enumeration increasing by 300 msec for numerosities in the subitizing range of 1–3, whilst showing no change in the counting range of 4–8. This is consistent with findings that dyscalculic children show impaired subitizing [35,48], and with the association between subitizing and number sense deficits seen in adult patients [61]. Previous research has demonstrated plasticity in subitizing performance in young adult video game players [72]. The present results suggest that this plasticity in numerical cognition can be harnessed by simpler computer games and be put to use for the remediation of dyscalculia.

Also consistent with the core deficit hypothesis was the large increase in children's subtraction accuracy (an average of 23% on non-rule problems), which suggests an improvement in the ability to manipulate or conceptualize quantities. This increase was not seen for addition, consistent with the idea that subtraction draws more strongly on quantity representation and manipulation than addition does [46]. More generally, as predicted, children showed little or no improvement on tasks which do not rely heavily on number sense; rule-based items in addition and subtraction as well as counting and transcoding. In counting there was no improvement seen in the 4–8 range of enumeration task, and although there was a slight improvement seen in the non-computerized task, this was in the more difficult aspects of the task, counting backwards and counting by twos or tens (which are arguably more a measure of calculation). There was only a small improvement seen in the non-computerized task of transcoding (8%). These last two tasks had a close to ceiling performance at initial testing, however this was not the case for the computerized enumeration task.

One task which showed a lack of improvement despite low initial performance was base-10 comprehension. We had no particular a priori hypothesis for this task, because there is no research on the relationship between base-10 comprehension and number sense, even in adults. In purely empirical terms these results cannot be seen as surprising because the software did not include two-digit numbers, or explicit training on the base-10 system. However, this does not exclude the possibility of an eventual benefit at a later stage (see transfer discussion below).

The dissociation between improved subtraction and unchanged addition requires further discussion. We have stressed the possibility that subtraction is more quantity-based than addition [46]. In adults, this is likely because few subtractions are memorized (thus they have to be solved by manipulating the quantities involved). In contrast, addition problems are often solved by verbal memory recall. Thus the failure of children to improve in addition could be seen as caused by a lower involvement of number sense in this process. However it should be noted that it is unknown to what extent dyscalculic children of this age solve addition problems by verbal memory.

An alternative possibility is that there was an initial qualitative difference in children's prior knowledge of subtraction vs. addition. In our sample, children seemed much less familiar with subtraction at the beginning of the study. Based on our observations during testing and a post-hoc error analysis, we found that in subtraction children made more errors which were conceptually rather than procedurally based; such as application of an incorrect rule (x-x = x, or x-0 = 0), adding instead of subtracting, or simply not knowing how to proceed to calculate a response (particularly not knowing how to use their fingers to do so). In contrast, all children were familiar with addition and the procedures of adding on their fingers or by verbal "counting up" (even if they did not do this fast or accurately). The types of errors made in addition indicated that children had grasped the general idea of adding. The majority of incorrect responses were actually non-responses due to children running out of time to execute slow finger counting procedures. The small amount of other errors were due to mis-counts, bridging errors (e.g. 8 + 6 = 4) or memory (e.g. 2 + 2 = 8) errors. Thus, improvement in subtraction may have resulted from improved conceptualization as a result of increased number sense. This may not have occurred for addition because children already had a reasonably solid concept of addition, and their difficulties lay more in the fast and accurate execution of counting procedures. The idea that the improvement seen in calculation tasks was due to conceptual rather than memory retrieval improvement fits with other findings that fact fluency in dyscalculics seems particularly resistant to remediation [73].

A third and final possibility is that training on particular problems failed to generalize to other problems. One important difference between the addition and subtraction tests was that the addition test included problems with a sum greater than 10 which were not trained in the software, whereas the subtraction task did not. The addition problems included in the software would have fallen into the "small" category only (sum < 10); however children's pre-remediation performance on "small normal" problems was already at 90% accuracy with a fairly fast response time of around 3 sec, thus there was not a large amount of room for improvement.

The preceding discussion brings up an important question for future research: To what extent does an improvement in number sense (or in its access via symbols) generalize to higher level mathematical tasks? This is an issue which is obviously of great importance for educators, because if a remediation of a number sense core deficit is to make a difference to dyscalculic children's performance in the classroom, this type of transfer must occur. Research in both the reading and mathematics domains suggests that transfer should take place, at least over developmental time. In the reading domain, training on the core processes of phonological discrimination and grapheme-phoneme correspondences generalizes to higher-level reading tasks [74-76]. In the mathematical domain, recent studies have shown that measures of number sense in the kindergarten years predict later performance in mathematics [e.g. [53]]. Earlier work by Sharon Griffin and colleagues [77,78] also suggests that training number sense in at-risk children in the kindergarten years can produce long lasting effects on mathematical performance as a whole.

The possible mechanisms for transfer necessarily remain speculative, because there is much we do not know about the development of numerical cognition, and in particular the role of number sense in aiding acquisition of higher level mathematical concepts and procedures. However, based on the hypothesis that number sense provides the semantics of number (or our internal "mental number line") [40,58], improving children's number sense and/or its access from symbolic information ought to, over time, produce general increases in comprehension of all aspects of mathematics. For instance, more accurate and faster access to number sense might be critical for conceptual understanding of addition and subtraction [56,77,78]. The attentional resources freed up by improved access to number semantics might also enhance children's ability to develop more efficient strategies such as breaking down problems into simpler steps, monitoring problem steps and solutions for errors, and relying less on concrete supports [36]. Facts might become easier and more efficient to memorize because of their higher semantic content [62]. Increases in accuracy in calculation might also reduce the likelihood of forming false associations in long term memory [36].

Assuming that transfer can occur, it is also crucial to know its timeframe. The current study suggests that transfer appears to be somewhat limited at the age of 7–9 and over a short period of two months. However, it is possible that better transfer would be seen at a longer post-training delay (and in fact this has been the case with phonemic awareness reading interventions [75]). There might also be a more propitious "developmental window" at a younger age, during which intervention is maximally effective.

### Achieving the instructional goals of the software

How well were the instructional goals of the software achieved? As presented in the accompanying article [1], these were 1) enhancing number sense, 2) cementing links between representations of number, and 3) conceptualizing and automatizing arithmetic.

We have seen that goals 1) and 2) appear to have been achieved to some degree. However, success on goal 3) was mixed. While the sub-goal of conceptualizing arithmetic may have been well achieved (see above discussion), the lack of improvement in addition suggests that that of automatizing arithmetic was not. This may have been partially due to the functioning of the software. Fact training was only present at the higher levels of difficulty, and the software took too long to reach these levels. The result was that addition and subtraction facts were only tested in a small percentage of trials (17% and 11% of game trials, or 72 and 43 problems per child on average, respectively). In future versions of the software, we plan to alter the program so that these higher levels can be reached much faster if the child is progressing well enough.

### Limitations of the study

The current results satisfy the goals of this initial study, which were to test for the presence of improvement and to identify sensitive pre-post measures. However several important limitations should be noted. Firstly, the study used a very small sample (n = 9). Secondly, in the absence of a control group, the observed effects could be due to specific classroom or home activity during the period of the study, or to an interaction between this activity and participation in the remediation program. Therefore our results should be taken with caution, as a first positive finding on the path towards establishing efficacy, and in need to be backed up by larger, controlled studies.

It should also be noted that the criteria for identifying dyscalculia are a subject of on-going debate and research, and vary widely from study to study. Thus with any study in this field it is difficult to say to what extent the sample is characteristic of the disorder. Our sample is clearly most parsimoniously described as having "mathematical learning difficulties". To what extent these children and their responses to intervention are similar to those selected using a stricter criteria remains to be established, as does whether different subtypes of dyscalculia might respond differently.

## Conclusion

In the first section of this paper, we described what is known about dyscalculia, and discussed the core deficit hypothesis, which proposes that dyscalculia is due to an underlying deficit in number sense or in its access via symbolic information. The pre and post testing results of our open-trial study showed that after using software designed to enhance number sense and its access via symbolic information for a total of 10 hours over a five week period, children made progress in several core areas of numerical cognition: number comparison, subitizing, and subtraction. These results are unlikely to be caused by general motivation or attentional effects because they were specific to particular tasks and to particular conditions within tasks.

We emphasize that our results are only a first step in the series of studies required to prove efficacy of the software. The inclusion of a control group is a critical next step, and the generality and duration of the effects found also need to be tested. Furthermore, much work remains to be done to establish to what extent basic training on number sense produces transfer to other higher level tasks, over what time period this occurs, the underlying brain mechanisms, and whether there is an optimal developmental time window during which intervention has the most impact.

## Competing interests

The author(s) declare that they have no competing interests.

## Authors' contributions

Collection and analysis of data for the open-trial study was carried out by AJW and SKR, with assistance from DC, SD and LC. All authors contributed intellectually to designing the open-trial study and interpreting its data, and all authors read and approved the final manuscript, which was prepared by AJW and SD.
